# Exercise as an airway clearance technique (ExACT) is not simply physical activity

**DOI:** 10.1177/17534666241292502

**Published:** 2024-11-08

**Authors:** Zoe L. Saynor, Don S. Urquhart

**Affiliations:** Department of Child Life and Health, University of Edinburgh, Edinburgh, UK; Department of Paediatric Respiratory and Sleep Medicine, Royal Hospital for Children and Young People, Edinburgh, UK; School of Health Sciences, Faculty of Environmental and Life Sciences, University of Southampton, Southampton, SO17 1BJ, UK; Wessex Cystic Fibrosis Unit, University Hospitals Southampton NHS Foundation Trust, Southampton, UK; National Institute for Health and Care Research, Southampton Biomedical Research Centre, University Hospital Southampton NHS Foundation Trust, UK

We read with interest the recent work by Blardone and colleagues,^1^ and wish to clarify key points regarding the potential role of Exercise as an Airway Clearance Technique (ExACT) in cystic fibrosis (CF). Whilst the authors correctly assert the notion that ACT can be replaced with physical activity is incorrect, their critique references our work and, we believe, reflects a misunderstanding of the terminology and specifics of ExACT.

Physical activity is broadly defined as any bodily movement requiring energy expenditure, whilst exercise is a structured, purposeful subcomponent of physical activity, typically involving aerobic and/or resistance training; both currently recommended standard clinical care for pwCF.^2^ ExACT, however, is distinct from both physical activity and general exercise. Developed through close collaboration with the CF community,^3^ ExACT is a bespoke exercise-based intervention,^3,4^ consisting of aerobic activity with vibration, that must be undertaken at a certain intensity, and specifically incorporates assessment breaths, huffs, and coughs to maximise airway clearance.^5^ Blardone and colleagues’ use of the term ‘physical activity’ in their critique of our work overlooks, and perhaps oversimplifies, these important distinctions.

Our work has never advocated replacing ACTs with general physical activity. Instead, we have sought to develop ExACT as an effective ACT, initially for people with CF. ExACT has been carefully defined by Delphi consensus,^3^ and subsequently refined (4) and tested in a pilot, randomised controlled trial. ExACT has been demonstrated to be feasible, acceptable and safe ()^5,6^ for pwCF who are stable on Elexacaftor/Tezacaftor/Ivacaftor (ETI), with a larger study to determine clinical and cost-effectiveness forthcoming.

Chest physiotherapy is often of suboptimal quality,^7^ and pwCF find it time-consuming^8^ and burdensome^9^; something Blardone *et al.*^1^ report persists for many in the era of highly effective modulator therapies, such as ETI. ExACT therefore offers promise in reducing the burden of ACTs for pwCF, and achieving more personalised ACT prescriptions,^2,10^ whilst also engaging them in structured exercise to improve and maintain health and well-being.^2^ In summary, therefore, whilst we agree that replacing traditional ACTs with physical activity is incorrect, our research suggests that ExACT is a bespoke modality and one that warrants further investigation.

## Declarations

**Ethics approval and consent to participate:** Not applicable.

**Consent for publication:** Not applicable.

**Availability of data and materials:** Not applicable.
